# Treatment effects and lip profile changes following premolars extraction treatment vs fixed functional treatment in Class II division 1 malocclusion: A randomized controlled clinical trial

**DOI:** 10.1590/2177-6709.28.2.e232140.oar

**Published:** 2023-05-19

**Authors:** Gagan Deep KOCHAR, Sanjay LONDHE, Sukhbir Singh CHOPRA, Sarvraj KOHLI, Virinder Singh KOHLI, Ashish KAMBOJ, Munish VERMA

**Affiliations:** 1 Government Dental Center (New Delhi, India).; 2 ADC R&R Hospital (New Delhi, India).; 3 Government Dental Center (Pune, India).; 4 Jabalpur Hospital (Jabalpur, India).; 5 Government Dental Center (Secunderabad, India).

**Keywords:** Extraction, Fixed functional appliance therapy, Randomized controlled trial

## Abstract

**Objective:**

The objective of this two-arm parallel randomized controlled trial was to evaluate the treatment effects and lip profile changes in skeletal Class II patients subjected to premolars extraction treatment *versus* fixed functional treatment.

**Methods:**

Forty six subjects fulfilling inclusion criteria were randomly distributed into Group PE (mean age 13.03±1.78 years) and Group FF (mean age 12.80±1.67 years) (n=23 each). Group PE was managed by therapeutic extraction of maxillary first premolars and mandibular second premolars, followed by mini-implant-supported space closure; and Group FF, by fixed functional appliance therapy. Skeletal, dental, and soft-tissue changes were analyzed using pre and post-treatment lateral cephalograms. Data obtained from this open label study was subjected to blind statistical analysis.

**Results:**

Extraction treatment resulted in greater increase of nasolabial angle (NLA: 3.1 [95% CI 2.08, 4.19], *p*<0.001), significant improvement of upper lip (UL-E line: -2.91 [95% CI -3.54, -2.28], *p*<0.001, UL-S line: -2.50 [95% CI -2.76, -2.24], *p*<0.001, UL-SnPog’: -2.32 [95% CI -2.90, -1.74], *p*<0.01) and lower lip position (LL-E line: -0.68 [95% CI -1.36, 0.00], *p*<0.01, LL-S line: -0.55 [95% CI -1.11, 0.02], *p*<0.01, and LL-SnPog’: -0.64 [95% CI -1.20, -0.07], *p*<0.01), lip thickness (UL thickness: 2.27 [95% CI 1.79, 2.75], *p*<0.001; LL thickness: 0.41 [95% CI -0.16, 0.97], *p*<0.01), upper lip strain (UL strain: -2.68 [95% CI -3.32, -2.04], *p*<0.001) and soft tissue profile (N’-Sn-Pog’: 2.68 [95% CI 1.87, 3.50], *p*<0.01). No significant difference was observed between the groups regarding skeletal changes in the maxilla and mandible, growth pattern, overjet, overbite, interincisal angle and soft tissue chin position (*p*>0.05). Premolar extraction treatment demonstrated significant intrusion-retraction of maxillary incisors, better maintenance of maxillary incisor inclination, and significant mandibular molar protraction; whereas functional treatment resulted in retrusive and intrusive effect on maxillary molars, marked proclination of mandibular anterior teeth, and significant extrusion of mandibular molars. Both treatment modalities had similar treatment duration. Implant failure was seen in 7.9% of cases, whereas failure of fixed functional appliance was observed in 9.09% of cases.

**Conclusions:**

Premolar extraction therapy is a better treatment modality, compared to fixed functional appliance therapy for Class II patients with moderate skeletal discrepancy, increased overjet, protruded maxillary incisors and protruded lips, as it produces better dentoalveolar response and permits greater improvement of the soft tissue profile and lip relationship.

## RESUMO

**Objetivo:**

O objetivo desse estudo randomizado controlado paralelo de dois braços foi avaliar os efeitos do tratamento e as mudanças no perfil labial em pacientes esqueléticos Classe II submetidos a tratamento com extração de pré-molares (EP) *versus* tratamento funcional fixo (FF).

**Métodos:**

Quarenta e seis indivíduos que preencheram os critérios de inclusão foram distribuídos aleatoriamente em Grupo EP (idade média 13,03±1,78 anos) e Grupo FF (idade média 12,80±1,67 anos) (n=23 cada). O grupo EP foi tratado com extração dos primeiros pré-molares superiores e segundos pré-molares inferiores, seguida de fechamento do espaço com ancoragem em mini-implantes; e o Grupo FF, com tratamento usando aparelhos funcionais fixos. As alterações esqueléticas, dentárias e de tecidos moles foram analisadas usando cefalogramas laterais pré e pós-tratamento. Os dados obtidos desse estudo aberto foram submetidos a análise estatística cega.

**Resultados:**

O tratamento com extrações resultou em maior aumento do ângulo nasolabial (ANL: 3,1 [IC 95% 2,08, 4,19], *p*<0,001), melhora significativa do lábio superior (Ls-Linha E: -2,91 [IC 95% -3,54, -2,28], *p*<0,001, Ls-Linha S: -2,50 [IC 95% -2,76, -2,24], *p*<0,001, Ls-SnPog’: -2,32 [IC 95% -2,90, -1,74], *p*<0,01) e posição do lábio inferior (Li-Linha E: -0,68 [IC 95% -1,36, 0,00], *p*<0,01, Li-Linha S: -0,55 [IC 95% -1,11, 0,02], *p*<0,01, e Li-SnPog’: -0,64 [IC 95% -1,20, -0,07], *p*<0,01), espessura dos lábios (espessura Ls: 2,27 [IC 95% 1,79, 2,75], *p*<0,001; espessura Li: 0,41 [IC 95% -0,16, 0,97], *p*<0,01), tensão do lábio superior (tensão Ls: -2,68 [IC 95% -3,32, -2,04], *p*<0,001) e perfil de tecidos moles (N’-Sn-Pog’: 2,68 [IC 95% 1,87, 3,50], *p*<0,01). Nenhuma diferença significativa foi observada entre os grupos quanto às alterações esqueléticas na maxila e mandíbula, padrão de crescimento, sobressaliência, sobremordida, ângulo interincisal e posição dos tecidos moles do mento (*p*>0,05). O tratamento com extração de pré-molares demonstrou significativa intrusão-retração dos incisivos superiores, melhor manutenção da inclinação dos incisivos superiores e protração significativa dos molares inferiores; enquanto o tratamento funcional resultou em efeito retrusivo e intrusivo nos molares superiores, proclinação acentuada dos dentes anteriores inferiores e extrusão significativa dos molares inferiores. Ambas as modalidades de tratamento tiveram duração de tratamento semelhante. A falha do mini-implante foi observada em 7,9% dos casos, enquanto a falha do aparelho funcional fixo foi observada em 9,09% dos casos.

**Conclusões:**

O tratamento com extração de pré-molares é uma modalidade de tratamento melhor do que os aparelhos funcionais fixos para pacientes Classe II com discrepância esquelética moderada, sobressaliência aumentada, incisivos superiores protruídos e lábios protruídos, pois produz melhor resposta dentoalveolar e permite maior melhora do perfil dos tecidos moles e relacionamento labial.

## INTRODUCTION

Class II malocclusion is a frequent condition, with a prevalence of around 30% of the patients seeking orthodontic treatment.^1^ This malocclusion has a variety of manifestations, which encompass dental, skeletal and soft tissue components. In Class II malocclusion, relative position of teeth affects the overjet, overbite and overlying soft tissues, which in turn influences the patients’ facial esthetics and quality of life.^2^

Depending on the underlying condition, multiple treatment strategies are available for the management of Class II malocclusion.^3^ Premolar extraction treatment routinely enables retraction of maxillary anterior teeth and optimization of overjet. There is a dichotomy among researchers regarding the effects of premolar extraction on esthetics of soft tissue profile. It is believed that reduction in dental volume secondary to extractions hampers the lip support.^4^ Though, extraction treatment has shown to be detrimental to facial profile and overbite, and strong arguments are being made against the use of this protocol^5^ - although existing scientific literature disavows these claims.^6^ ^-^ ^9^

Treatment by both fixed and removable functional appliances is effective in correcting the Class II malocclusion.^10^ The masking of underlying skeletal discrepancy by functional treatment is mainly due to transient rather than additional bony growth.^11^ Orthopedic management primarily produces dentoalveolar changes, with limited skeletal effects. Contradictory results regarding the effect of fixed functional appliance on soft tissues have been reported in literature.^12^ ^,^ ^13^

The last decade has witnessed a steady upsurge in the interest for facial esthetics. Therefore, soft tissues changes following orthodontic treatment are given more importance than ideal occlusion. Presently, data is lacking on the relationship of soft tissue profile changes and both treatment modalities, with and without extractions.^14^ The present study endeavors to contribute to the existing knowledge base. This prospective randomized clinical trial was conducted with the aim to compare the treatment effects and lip profile changes in skeletal Class II patients subjected to premolar extractions and those treated with fixed functional appliance therapy. The null hypothesis was that there would be no statistically significant difference in treatment outcome and profile change of patients with Class II malocclusion treated with extraction of premolars and those treated with fixed functional appliance therapy.

## MATERIAL AND METHODS

### TRIAL DESIGN AND SETTING

This study was conducted as a two-arm parallel randomized controlled trial. There were no changes after commencement of the study. Subjects for the trial were recruited and treated in the outpatient clinic of Department of Orthodontics of a tertiary care centre, from 2016 to 2019. 

### SAMPLE SIZE CALCULATION

The sample size was calculated using G*Power software v. 3.0.8 (Universität Kiel, Germany), based on a significance value of 0.05 and a power of 80%. Power analysis determined that a minimum of 18 subjects were required in each group to demonstrate a significant change of upper lip to H line of 1.25mm, which was similar to previously published studies.^15^ Considering certain amount of dropouts, a greater number of subjects was selected.

### PARTICIPANTS AND ELIGIBILITY CRITERIA

**Figure 1:**
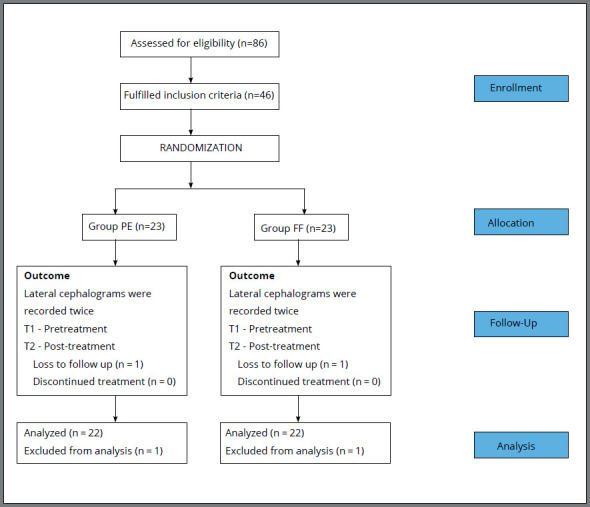
Patient selection and follow up flow chart.

Patient selection and follow-up were conducted in accordance with CONSORT guidelines (Fig 1). Recruitment for the trial was conducted from September 2016 to January 2017. Then, 86 patients were screened and 46 (Male/Female - 24/22) skeletal Class II patients of mixed Indian population were enrolled in the study (Table 1). Informed consent was obtained from all the subjects, prior to recruitment for this study. The research proposal was approved by the research ethics committee of the respective institute (Number - 145/200/2071). 

**Table 1:** Descriptive statistics of the participants.

Sample size	Group PE	23
	Group FF	23
Age (years)	Group PE	13.03 ± 1.78
	Group FF	12.80 ± 1.67
Gender	Group PE	Male = 13, Female = 10
	Group FF	Male = 11, Female = 12
Treatment duration (years)	Group PE	2.22 ± 0.28
	Group FF	2.08 ± 0.24

* Group PE (Premolar extraction treatment). * Group FF (Fixed Functional appliance therapy).

Selection criteria included skeletal Class II malocclusion, at least – Class II molar and canine relationship, Cervical Vertebrae Maturity Index (CVMI) confirming circumpubertal phase of skeletal development, average or horizontal growth pattern, no crowding in the maxillary arch, minimal crowding (<3mm) in the mandibular arch, and overjet greater than 5mm but less than 8mm. Patients with history of previous orthodontic treatment, systemic disorders, or syndromic cases were excluded from the study. 

Finally, 17 subjects presented with bilateral Class II molar relationship, 14 had bilateral – Class II molar, 15 had Class II molar on one side and – Class II molar on the other side. 

### RANDOMIZATION

Following recruitment and consent, a random list of all the subjects was created online (https://www.random.org/) by an individual who was not involved in the trial. The allocation sequence was concealed from research personnel and assessor, using sealed opaque envelopes, which were numbered sequentially and were chosen by the patients. Subjects were randomly allocated in 1:1 ratio into the two arms of this study i.e.: Group PE - Premolar extraction treatment (mean age = 13.03±1.78 years) and Group FF - Fixed Functional Appliance Therapy (mean age = 12.80±1.67 years), with 23 subjects each. There was no restricted randomization.

### INTERVENTION

Clinical management in Group PE included extraction of maxillary first premolars and mandibular second premolars. Since subjects had varying pretreatment molar relationship, extractions were carried out in the mandibular arch in order to achieve similar molar relation bilaterally at the end of the treatment. The 0.018 x 0.028-in slot brackets (Roth prescription) were bonded, and levelling and alignment was carried out using 0.016-in nickel-titanium (NiTi) archwire, followed by 0.016 x 0.022-in NiTi. Working archwire (0.016 x 0.022-in stainless steel) was left *in situ* for a period of four weeks prior to the commencement of retraction. Anchorage reinforcement was done using maxillary mini-implants (8-mm long, 1.3-mm diameter, Abso Anchor, Dentos Inc, Daegu, Korea) placed into interradicular bone between the second premolars and the first molars bilaterally, and mandibular mini-implants (6-mm long, 1.2-mm diameter, Abso Anchor) placed into interradicular bone between the canine and first premolars bilaterally, under local anesthesia. After ensuring the primary stability, implants were immediately loaded. Space closure was done by sliding mechanics using elastic e-chain (Memory Chain, Short, American Orthodontics, Sheboygan, WI, USA) and force of 150g, quantified using a force gauge (Dentaurum, Ispringen, Germany), was applied bilaterally. After closure of extraction spaces, mini-implants were removed in all the subjects.

In Group FF, similar bracket prescription was used and working archwire was 0.017 x 0.025-in stainless steel. Forsus™ (Fatigue Resistant Device, L-pin Spring Module, 3M), a fixed functional appliance, was used during the functional phase. The appliance selection and stepwise activation using 1.5-mm split rings was done according to the manufacturer’s instructions. Whenever more than one split ring was required for activation, the next size push rod was used. Average duration of the functional phase was 6.95±1.53 months. Functional phase continued until bilateral Class I molar and canine relation, optimized overbite and overjet were achieved. Following, non-activated appliance (i.e. spring module not compressed by the push rod as the patient bites down) was left *in-situ* for one appointment interval. After removal of the Forsus™, post-functional orthodontics was carried out using customized 0.016-in stainless steel mandibular archwire and Class II elastics 5/16-in, 2.5oz (U3s?L6s) and triangular elastics 3/16-in, 3.5oz (U3, U5s?L5s).

All the subjects were scheduled every three weeks during the course of treatment. Post-debonding, patients were followed periodically during retention. 

### OUTCOME

**Figure 2:**
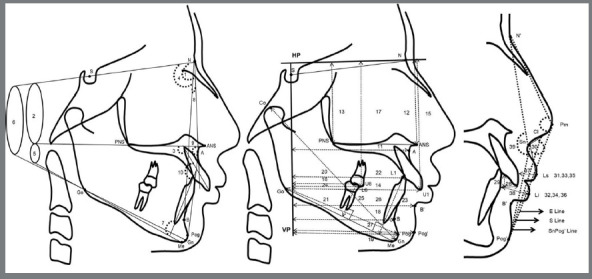
Angular measurement (degrees) and linear measurements (mm) used in the study: (1) SNA; (2) SN - ANS PNS; (3) U1 - ANS PNS; (4) SNB; (5) FMA; (6) SN -GoGn; (7) IMPA; (8) ANB; (9) N - A - Pog; (10) Interincisal angle; (11) A - VP; (12) N - ANS; (13) N - PNS; (14) U1 - VP; (15) U1 - HP; (16) U6 - VP; (17) U6 - HP; (18) B - VP; (19) Pog - VP; (20) CoGn; (21) GoPog; (22) L1 - VP; (23) L1 - GoMe; (24) L6 - VP; (25) L6 - GoMe; (26) B’ - VP; (27) Pog’ - VP; (28) Overjet; (29) Overbite; (30) NLA; (31) UL - E line; (32) LL - E line; (33) UL - S line; (34) LL - S line; (35) UL - SnPog’ (36) LL - SnPog’; (37) UL thickness; (38) LL thickness; (39) N’ - Sn - Pog’. (Refer Supplementary Table 1 and Supplementary Table 2 for definitions of landmarks and cephalometric parameters).

Primary outcome was to test the hypothesis that there would be no statistically significant difference in soft tissue and profile change of patients with Class II malocclusion treated with premolars extraction and those treated with fixed functional treatment. Lateral cephalograms (Pretreatment - T1 and Post-treatment - T2) were recorded, to monitor treatment changes. Skeletal (n=14), dental (n=13) and soft tissue (n= 3) measurements were recorded, to determine the changes produced by the treatment (Fig 2) (Supplementary Tables 1 and 2). Horizontal plane - HP (7° to SN plane) and vertical plane - VP (plane perpendicular to HP passing though Sella) were used as reference planes, similar to previous studies.^16^

**Supplementary Table 1:** Landmarks used for cephalometric evaluation.

No	Landmark	Definition
1.	N	Most anterior point of frontonasal suture in the median plane
2.	S	Midpoint of the hypophyseal fossa
3.	A	Deepest midline point in the curved bony outline from the base to the alveolar process of maxilla
4.	ANS	Tip of the bony anterior nasal spine in the median plane
5.	PNS	Intersection of the continuation of anterior wall of the pterygopalatine fossa and floor of the nose
6.	B	Most posterior point in the outer contour of mandibular alveolar process in the median plane
7.	Pog	Most anterior point of the bony chin
8.	Gn	Most anteroinferior point of the bony chin
9.	Me	Most inferior point of the bony chin
10.	Go	Most posterior and inferior point on outline of mandibular angle
11.	U1	Incisal tip of most prominent maxillary incisor
12.	U6	Mesiobuccal cusp tip of maxillary molar
13.	L1	Incisal tip of most prominent mandibular incisor
14.	L6	Mesiobuccal cusp tip of mandibular molar
15.	N’	The point of greatest concavity in the midline between the forehead and the nose
16.	Prn	Most anterior point on the nose
17.	Cl	Columella
18.	Sn	Point at which the columella merges with the upper lip in the midsagittal plane
19.	Ls	Most anterior point on upper lip
20.	Li	Most anterior point on lower lip
21.	B’	Most posterior point on the soft tissue chin in the midsagittal plane
22.	Pog’	Most anterior point on the soft tissue chin in the midsagittal plane

**Supplementary Table 2:** Cephalometric parameters.

No	Landmark	Definition
1.	SNA	The inferior posterior angle between SN and NA
2.	SN - ANS PNS	Angle formed between SN and ANS PNS
3.	U1 - ANS PNS	Angle formed between long axis of most prominent maxillary central incisor and ANS PNS
4.	SNB	The inferior posterior angle between SN and NB
5.	FMA	Angle formed between ANS PNS and GoMe
6.	SN - GoGn	Angle formed between SN and GoGn
7.	IMPA	Angle formed between long axis of most prominent mandibular central incisor and GoMe
8.	ANB	Angle formed by the intersection of NA and NB
9.	N - A - Pog	Angle formed between NA and APog
10.	Interincisal angle	Angle formed between long axis of most prominent maxillary central incisor and mandibular central incisor
11.	A - VP	Perpendicular distance between point A and VP
12.	N - ANS	Perpendicular distance between ANS and HP
13.	N - PNS	Perpendicular distance between PNS and HP
14.	U1 - VP	Perpendicular distance between incisal tip of U1 and VP
15.	U1 - HP	Perpendicular distance between incisal tip of U1 and HP
16.	U6 - VP	Perpendicular distance between incisal tip of U6 and VP
17.	U6 - HP	Perpendicular distance between incisal tip of U6 and HP
18.	B - VP	Perpendicular distance between point B and VP
19.	Pog - VP	Perpendicular distance between Pog and VP
20.	CoGn	Linear distance between Co and Gn
21.	GoPog	Linear distance between Go and Pog
22.	L1 - VP	Perpendicular distance between incisal tip of L1 and VP
23.	L1 - GoMe	Perpendicular distance between incisal tip of L1 and GoMe plane
24.	L6 - VP	Perpendicular distance between incisal tip of L6 and VP
25.	L6 - GoMe	Perpendicular distance between incisal tip of L6 and GoMe plane
26.	B’ - VP	Perpendicular distance between B’ and VP
27.	Pog’ - VP	Perpendicular distance between Pog’ and VP
28.	Overjet	Distance between the incisal edges of the maxillary and mandibular central incisors, parallel to the occlusal plane
29.	Overbite	Distance between the incisal edges of the maxillary and mandibular central incisors, perpendicular to the occlusal plane
30.	NLA	Angle formed by Cl, Sn, and Ls
31.	UL - E line	Perpendicular distance between Ls and E line (line from Prn to Pog’)
32.	LL - E line	Perpendicular distance between Li and E line
33.	UL - S line	Perpendicular distance between Ls and S line (line from Cl to Pog’)
34.	LL - S line	Perpendicular distance between Li and S line
35.	UL - SnPog’ line	Perpendicular distance between Ls and SnPog’ line (line from Sn to Pog’)
36.	LL - SnPog’ line	Perpendicular distance between Li and SnPog’ line
37.	UL thickness	Shortest distance between Ls and the most prominent labial point of the maxillary incisor
38.	LL thickness	Shortest distance between Li and the most prominent labial point of the mandibular incisor
39.	N’-Sn-Pog’	Angle formed between N’Sn and SnPog’
40.	Lip strain	The difference between basic upper lip thickness (linear distance from 3 mm below A-point to Sn) and upper lip thickness

The secondary outcome included skeletal and dental treatment changes, overall treatment duration and overall failure rate of implants / fixed functional appliance. 

### INTERIM ANALYSES AND STOPPING GUIDELINES

Not applicable.

### BLINDING

It was not feasible to blind for the clinical procedures. Blinding was done for statistical analysis only.

## ERROR OF THE METHOD AND STATISTICAL ANALYSIS

Ten cephalograms were randomly selected and retraced after a two-weeks interval, to determine the intra and inter-operator reliability. Allocation of all the traced cephalograms was done by a random list created online (https://www.random.org/). The reliability of recorded measurements was determined using intra-class correlation coefficient (ICC): ICC closer to 1 indicated highly reliable measurement. 

The data collected were compiled in Excel spreadsheet (Microsoft, Redmond, Wash) and transferred to SPSS v. 22.0 software (SPSS Inc., Chicago, IL, USA). Normality of the data distribution was verified using the Shapiro-Wilk test, and was found to be non-significant for all the variables. Gender comparison was performed using the Mann-Whitney U test. Age and treatment duration of the groups were compared using Student *t*-test. Means and standard deviations of pretreatment (T1) and post-treatment (T2) cephalometric parameters were calculated, and Student *t*-test was used to compare the cephalometric data of the two groups. Values of *p*< 0.05 were considered statistically significant.

## RESULTS

### OUTCOME

**Figure 3:**
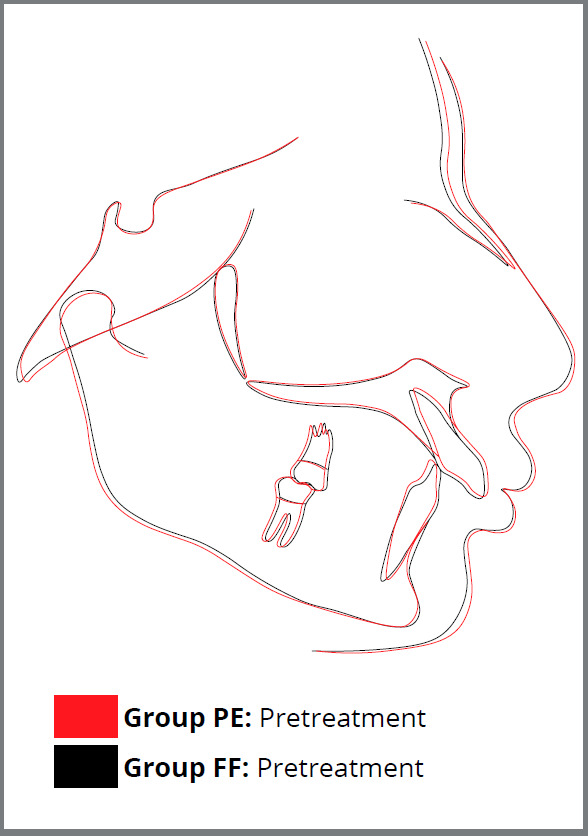
Superimposition of average cephalometric tracings of Group PE (Premolar extraction treatment) and Group FF (Fixed functional appliance therapy) at T1.

The mean age of the subjects of Groups PE and FF at the start of study was 13.03±1.78 and 12.80±1.67 years, respectively. Average duration of premolar extraction treatment was 2.22±0.28 years, and of fixed functional treatment was 2.08±0.24 years, with no significant difference between the two groups (Table 1). Subjects of both groups demonstrated no difference in distribution in terms of age, gender and cephalometric parameters investigated (*p* > 0.05) (Fig 3).

ICC of intra-examiner and inter-examiner measurements was 0.96 and 0.92, respectively, depicting highly reliable measurements. The Student *t*-tests comparing the pretreatment parameters and the treatment changes between the groups are presented in Tables 2 to 5.

**Table 2:** Comparison of cephalometric parameters of Group PE (Premolar extraction treatment) and Group FF (Fixed Functional appliance therapy) at T1.

Parameter	Group PE		Group FF		P value
	Mean	SD	Mean	SD	
Maxillary measurements					
SNA (degrees)	82.09	2.91	81.77	2.22	NS
A - VP (mm)	77.18	3.55	78.59	3.83	NS
SN - ANS PNS (degrees)	6.45	2.04	6.55	2.39	NS
N - ANS (mm)	51.27	3.48	50.45	3.22	NS
N - PNS (mm)	50.41	3.00	49.68	3.12	NS
Maxillary Dental relationship					
U1 - VP (mm)	83.09	3.57	81.82	3.17	NS
U1 - HP (mm)	77.41	3.16	78.82	3.25	NS
U6 - VP (mm)	40.41	3.65	41.05	4.01	NS
U6 - HP (mm)	70.23	3.02	71.73	3.04	NS
U1 - ANS PNS (degrees)	119.41	4.14	116.59	4.60	NS
Mandibular measurements					
SNB (degrees)	75.95	2.73	75.05	1.76	NS
B - VP (mm)	70.64	4.32	71.68	5.76	NS
Pog - VP (mm)	73.32	4.62	74.32	5.64	NS
CoGn (mm)	94.14	7.58	95.73	7.37	NS
GoPog (mm)	68.27	5.33	69.77	6.29	NS
FMA (degrees)	22.68	3.12	21.91	3.62	NS
SN - GoGn (degrees)	30.05	3.90	29.50	2.87	NS
Mandibular Dental relationship					
IMPA (degrees)	94.73	4.10	95.68	5.83	NS
L1 - VP (mm)	76.95	5.99	77.32	5.09	NS
L1 - GoMe (mm)	35.41	5.04	37.64	4.61	NS
L6 - VP (mm)	36.18	4.34	37.05	5.27	NS
L6 - GoMe (mm)	30.86	5.50	29.45	6.03	NS
Maxillomandibular Skeletal Relationship					
ANB (degrees)	6.14	1.36	6.73	1.78	NS
N - A - Pog (degrees)	170.32	3.33	169.14	3.21	NS
Maxillomandibular Dental Relationship					
Overjet (mm)	6.73	1.32	7.09	1.54	NS
Overbite (mm)	5.23	1.34	5.86	1.61	NS
Interincisal angle (degrees)	121.73	4.43	120.68	5.09	NS
Soft tissue measurements					
NLA (degrees)	93.41	5.84	94.68	5.24	NS
UL - E line (mm)	2.27	1.91	1.32	1.70	NS
LL - E line (mm)	0.73	1.75	0.55	2.24	NS
UL - S line (mm)	4.18	1.47	3.45	1.63	NS
LL - S line (mm)	2.64	1.68	2.36	1.53	NS
UL - SnPog’ (mm)	6.64	1.53	5.86	2.10	NS
LL - SnPog’ (mm)	4.45	1.01	4.32	1.13	NS
UL thickness (mm)	13.05	1.65	13.50	1.60	NS
UL strain (mm)	2.09	1.11	2.23	1.23	NS
LL thickness (mm)	16.23	2.25	16.91	2.27	NS
N’ - Sn - Pog’ (degrees)	155.18	5.67	153.36	5.19	NS
B’ - VP (mm)	79.73	4.42	80.09	3.75	NS
Pog’ - VP (mm)	82.45	4.82	83.73	5.72	NS

NS - Not Significant.

**Table 3:** Comparison of soft tissue changes (T2-T1) between Group PE (Premolar extraction treatment) and Group FF (Fixed Functional appliance therapy).

Parameter	Group PE			Group FF			P value
Soft tissue measurements	Mean	SD	95% CI	Mean	SD	95% CI	
NLA (degrees)	3.14	2.05	(2.08, 4.19)	0.59	1.89	(-0.38, 1.56)	**
UL - E line (mm)	-2.91	1.23	(-3.54, -2.28)	-1.50	1.34	(-2.19, -0.81)	**
LL - E line (mm)	-0.68	1.32	(-1.36, 0.00)	0.45	1.79	(-0.47, 1.38)	*
UL - S line (mm)	-2.50	0.51	(-2.76, -2.24)	-1.27	1.32	(-1.95, -0.60)	**
LL - S line (mm)	-0.55	1.10	(-1.11, 0.02)	0.36	1.26	(-0.28, 1.01)	*
UL - SnPog’ (mm)	-2.32	1.13	(-2.90, -1.74)	-1.36	1.05	(-1.90, -0.82)	*
LL - SnPog’ (mm)	-0.64	1.09	(-1.20, -0.07)	0.36	1.09	(-0.20, 0.93)	*
UL thickness (mm)	2.27	0.94	(1.79, 2.75)	1.09	1.06	(0.54, 1.64)	**
UL strain (mm)	-2.68	1.25	(-3.32, -2.04)	-1.18	1.05	(-1.72, -0.64)	**
LL thickness (mm)	0.41	1.10	(-0.16, 0.97)	-0.32	0.95	(-0.80, 0.17)	*
N’ - Sn - Pog’ (degrees)	2.68	1.59	(1.87, 3.50)	1.27	1.08	(0.72, 1.83)	*
B’ - VP (mm)	0.27	1.03	(-0.26, 0.80)	0.95	1.33	(0.27, 1.64)	NS
Pog’ - VP (mm)	0.32	1.17	(-0.28, 0.92)	1.09	1.74	(0.19, 1.99)	NS

NS = Not Significant, * p < 0.05, ** p < 0.001.

**Table 4:** Comparison of skeletal changes (T2-T1) between Group PE (Premolar extraction treatment) and Group FF (Fixed Functional appliance therapy).

Parameter	Group PE			Group FF			P value
	Mean	SD	95% CI	Mean	SD	95% CI	
Maxillary measurements							
SNA (degrees)	-0.77	1.11	(-1.34, -0.20)	-1.14	1.04	(-1.67, -0.60)	NS
A - VP (mm)	-0.68	1.21	(-1.30, -0.06)	-1.36	1.50	(-2.13, -0.59)	NS
SN - ANS PNS (degrees)	0.41	1.05	(-0.13, 0.95)	0.36	1.05	(-0.18, 0.90)	NS
N - ANS (mm)	0.36	1.26	(-0.28, 1.01)	0.32	1.17	(-0.28, 0.92)	NS
N - PNS (mm)	0.45	1.26	(-0.19, 1.10)	0.36	1.18	(-0.24, 0.97)	NS
Mandibular measurements							
SNB (degrees)	0.55	1.22	(-0.08, 1.17)	1.23	1.15	(0.64, 1.82)	NS
B - VP (mm)	0.59	1.30	(-0.08, 1.26)	1.41	1.59	(0.59, 2.23)	NS
Pog - VP (mm)	0.41	1.10	(-0.16, 0.97)	1.14	1.36	(0.44, 1.83)	NS
CoGn (mm)	1.00	1.75	(0.10, 1.90)	2.09	1.82	(1.15, 3.03)	NS
GoPog (mm)	0.82	1.26	(0.17, 1.47)	1.68	1.64	(0.84, 2.53)	NS
FMA (degrees)	-0.41	1.56	(-1.21, 0.39)	0.32	1.62	(-0.51, 1.15)	NS
SN - GoGn (degrees)	-0.64	2.04	(-1.68, 0.41)	0.36	1.99	(-0.66, 1.39)	NS
Maxillomandibular Skeletal Relationship							
ANB (degrees)	-1.27	1.67	(-2.18, -0.46)	-2.27	1.08	(-2.99, -1.74)	NS
N - A - Pog (degrees)	1.23	1.31	(0.56, 1.90)	2.00	1.54	( 1.21, 2.79)	NS

NS = Not Significant, * p < 0.05, ** p < 0.001.

**Table 5:** Comparison of dental changes (T2-T1) between Group PE (Premolar extraction treatment) and Group FF (Fixed Functional appliance therapy).

Parameter	Group PE			Group FF			P value
	Mean	SD	95% CI	Mean	SD	95% CI	
Maxillary Dental relationship							
U1 - VP (mm)	-6.91	2.00	(-7.94, -5.88)	-5.18	1.87	(-6.14, -4.22)	*
U1 - HP (mm)	-0.95	2.01	(-1.99, 0.08)	0.91	1.54	(0.12, 1.70)	*
U6 - VP (mm)	0.59	1.14	(0.00, 1.18)	-1.86	1.39	(-2.58, -1.15)	**
U6 - HP (mm)	0.73	1.16	(0.13, 1.32)	0.09	1.02	(-0.43, 0.61)	NS
U1 - ANS PNS (degrees)	-4.09	0.75	(-4.48, -3.71)	-5.73	1.70	(-6.60, -4.86)	**
Mandibular Dental relationship							
IMPA (degrees)	2.05	1.56	(1.24, 2.85)	5.23	1.97	(4.21, 6.24)	**
L1 - VP (mm)	1.64	1.43	(0.90, 2.37)	4.14	1.49	(3.37, 4.90)	**
L1 - GoMe (mm)	-0.23	1.51	(-1.00, 0.55)	-1.14	1.25	(-1.78, -0.50)	*
L6 - VP (mm)	4.82	1.30	(4.15, 5.48)	3.18	1.05	(2.64, 3.72)	*
L6 - GoMe (mm)	1.41	1.26	(0.76, 2.06)	2.45	1.82	(1.52, 3.39)	*
Maxillomandibular Dental Relationship							
Overjet (mm)	-4.23	0.81	(-4.65, -3.81)	-4.73	0.83	(-5.15, -4.30)	NS
Overbite (mm)	-2.64	1.00	(-3.15, -2.12)	-3.27	1.49	(-4.04, -2.51)	NS
Interincisal angle (degrees)	3.23	1.66	(2.37, 4.08)	4.27	1.75	(3.37, 5.17)	NS

NS = Not Significant, * p < 0.05, ** p < 0.001.

Both treatment mechanics were compared for 40 cephalometric parameters. Apart from the skeletal changes in the maxilla and mandible, growth pattern, overjet, overbite, interincisal angle and soft tissue chin position (*p* > 0.05), all other parameters demonstrated significant difference. 

**Figure 4:**
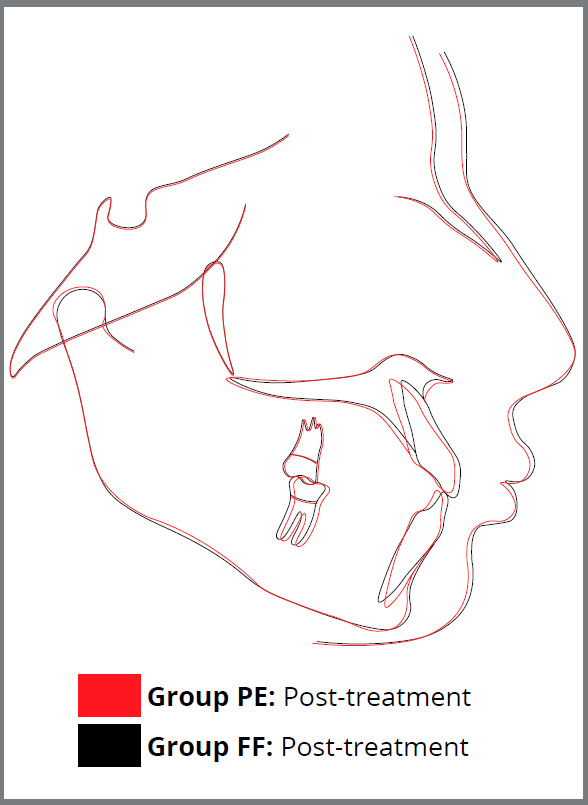
Superimposition of average cephalometric tracings of Group PE (Premolar extraction treatment) and Group FF (Fixed Functional appliance therapy) at T2.

With regard to the soft tissue changes (Fig 4), treatment effects were evident on almost all cephalometric parameters (Table 3), except for B’-VP and Pog’-VP (*p* > 0.05). Group PE had significant increase of nasolabial angle (NLA, *p*= 0.0001), greater retraction of upper lip (UL-E line, *p*= 0.0007, UL-S line, *p*= 0.0002 and UL-SnPog’, *p*= 0.006) and lower lip position (LL-E line, *p*= 0.02, LL-S line, *p*= 0.02 and LL-SnPog’, *p*= 0.04), significant increase of upper and lower lip thickness (UL thickness, *p*= 0.0003; LL thickness, *p*= 0.02), significant improvement of upper lip strain (UL strain, *p*= 0.0009) and significant improvement of soft tissue profile (N’-Sn-Pog’, *p*= 0.001).

With regard to the skeletal changes (Table 4), both treatment modalities had retrusive effect on the maxilla along sagittal plane, but differences were not significant (SNA; A-VP, *p* > 0.05). Difference in improvement of mandibular sagittal discrepancy following both treatment strategies was not significant (SNB; B-VP; Pog-VP; Co-Gn; Go-Pog, *p* > 0.05). Maxillomandibular skeletal relationship improved with both treatment mechanics, but differences werenot significant (ANB; NA-Pog, *p* > 0.05). 

Along the vertical plane, effect of both protocols had no significant difference (SN-ANS PNS; N-ANS; N-PNS; FMA; SN-GoGn, *p*> 0.05). 

With regard to the dentoalveolar changes (Table 5), premolar extraction treatment demonstrated significant intrusion-retraction of maxillary incisors (U1-VP, *p*= 0.005; U1-HP, *p*= 0.001), better maintenance of maxillary incisor inclination (U1-ANS PNS, *p*= 0.0001) and significant mandibular molar protraction (L6-VP, *p*= 0.004); whereas functional treatment produced retrusive as well as intrusive effect on maxillary molars (U6-VP, *p*= 0.0001; U6-HP, *p*= 0.06), marked proclination of mandibular anterior teeth (IMPA, *p*= 0.0005; L1-VP, *p*= 0.0001) and significant extrusion of mandibular molars (L6-GoMe, *p*= 0.03). 

### APPLIANCE / IMPLANT FAILURE

The implant failure rate was 7.9% - i.e., only 7 of the total implants placed became loose. Failure of the functional appliance was reported in 3 cases: One of the subjects had recurrent breakages of the functional appliance spring and was thus excluded from the present study.

## DISCUSSION

Successful orthodontic treatment is not just about establishing a static as well as dynamic occlusal relationship, but also to achieve an esthetic soft tissue profile.^17^ Over the years, an enhanced interest for facial esthetics and the changes in the soft tissues secondary to the orthodontic treatment are being widely researched across the globe. Up to the present date, there is no consensus among researchers regarding the outcomes of premolar extraction therapy *versus* fixed functional appliance in skeletal Class II patients. 

In the present study, there was a possibility of treatment outcome being influenced by associated growth. To overcome this, use of untreated Class II controls was advocated by Stahl et al.^18^ Due to ethical concerns, it is difficult to have such a control group nowadays. Though, some researchers have used data from growth studies as standard control, however secular growth trends question the validity of this data.^19^

Ideally, the subjects of a trial should demonstrate no difference in characteristics prior to the treatment, as phenotypic differences may influence the response to growth and treatment mechanics.^20^ Closely matched groups of mixed Indian population with no difference in distribution in terms of age, gender and cephalometric parameters - i.e., moderate skeletal discrepancy, increased overjet, protruded maxillary incisors- were selected and investigated in the present study (Tables 1 and 2). 

Severity of sagittal molar relationship directly influences the amount of retraction in Class II cases; hence, considering it during a trial was proposed in a systematic review.^21^ In the present trial, subjects with at least – Class II molar and canine relationship were included.

Soft tissue response secondary to aging and treatment assists clinician in planning the best suited treatment for the patient. Bishara et al^22^ have advocated lip protrusion as one of the critical factors in extraction cases. Patients of Group FF had lip protrusion just as those of Group PE at pretreatment. Compared to the functional treatment, patients subjected to premolars extraction demonstrated significant retrusion of lips (Table 3). 

Change in lip projection of Group PE (UL-E line, -2.91mm; LL-E line, -0.68mm) and Group FF (UL-E line, -1.50mm; LL-E line, 0.45mm) were in partial agreement with Janson et al^23^, Weyrich and Lisson^9^ , who reported an average retraction of -0.44mm and -2.17mm, respectively, of lower lip in patients subjected to functional treatment. However, the present findings were in agreement with Upadhyay et al^24^, who reported similar effects but with greater quantifications of lower lip changes. In the present study, substantial change in lip projection is attributable to significant implant-supported *en-masse* retraction of anterior teeth. 

Lee et al^25^ emphasized that lip strain and lip thickness must be evaluated based on dental inclination, to obtain balance in the perioral muscle activity. The effect of both protocols on lip thickness and lip strain has also been evaluated. In Group PE, upper lip thickness increased by 2.27mm, while upper lip strain reduced by 2.68mm; whereas in Group FF the quantifications were 1.09mm and -1.18mm, respectively, indicating greater changes following *en-masse* retraction, thus agreeing with previous evidence.^26^ The reduction in lip strain may be attributable to the recovery of upper lip thickness following dental retraction. Lower lip demonstrated an increase in thickness in Group PE (0.41mm) and reduction in Group FF (-0.32mm). 

Group PE demonstrated statistically significant change of nasolabial angle (3.14±2.05, *p*= 0.0001), with the variation ranging from 2.08 to 4.19 degrees; whereas in Group FF, variation ranged from -0.38 to 1.56 degrees, which is in consonance with previous study,^27^ but in disagreement with Upadhyay et al,^24^ who reported change of 12^o^, and Weyrich and Lisson,^9^ who reported an insignificant increase in subjects treated with four premolars extraction. In Class II division 1 cases presenting with minimal crowding, the maxillary anterior dentition must be distalized through entire premolar extraction space. In the present study, substantial change in nasolabial angle is attributable to retraction of upper lip secondary to significant implant-supported *en-masse* retraction of maxillary anterior teeth. 

Improvement of facial esthetics is usually the primary objective of patients undergoing treatment for skeletal Class II malocclusion.^28^ In Group FF, soft tissue profile improved by 1.27^o^, thus agreeing with previous evidence.^10^ Several factors such as age, gender, phenotypic presentation, treatment mechanics and anchorage devices may influence the response of soft tissue profile to treatment.^21^ It was interesting to note that despite insignificant skeletal changes, extraction protocol demonstrated significant improvement of soft tissue profile (Table 3), contrary to Kinzinger et al,^29^ who reported insignificant change of skeletal, as well as soft tissue profile. Variations in findings may be attributable to the use of extraoral anchorage in their study. Significant soft tissue profile change in Group PE was mainly attributable to dentoalveolar changes. In Groups FF and PE, B’ moved anteriorly by 0.95mm and 0.27mm, respectively; whereas Pog’ moved forward by 1.09mm and 0.32mm, respectively - not differing from the changes reported by Franchi et al.^30^

Clinical significance of the statistically significant findings of the present study could not be differentiated, due to the dichotomy in available literature. Cozza et al^31^ emphasized that statistically significant changes of less than two units may not be clinically significant, whereas Bowman et al^32^ reported improved facial esthetics perception by both dentists and layperson with mere 1.8-mm reduction of lip protrusion.

With regard to the skeletal changes, both treatment mechanics had a retrusive effect on the maxillary sagittal growth, resulting in the remodeling of point ‘A’, but the differences were not significant.

Compared to the premolar extraction treatment, functional fixed therapy demonstrated slightly larger sagittal growth of the mandible, but posttreatment differences were not statistically significant. Although improvement of maxillomandibular skeletal relationship was greater with functional treatment, compared to premolar extraction treatment, the differences were not significant. Similar changes were referred by a previous study.^24^ In Group FF, CoGn increased by 2.09mm and GoPog, by 1.68mm -no greater than the changes reported by Vaid et al^33^ -; whereas in Group PE, CoGn and GoPog increased by 1.00mm and 0.82mm, respectively. 

Effects of both treatment modalities on the vertical plane did not differ significantly among treatment groups. However, functional fixed treatment resulted in clockwise rotation of both jaws, whereas counter-clockwise mandibular rotation was observed in the subjects who underwent premolar extractions. Similar changes were reported by previous studies.^29^ However, the present findings were not in agreement with those of Basciftci and Usumez^34^, as they reported an increase of SN-GoGn in Class II extraction cases. Variations may be attributable to clinicians’ experience. In the present study, the reduction of Sn-GoGn and FMA following extraction of premolars may be attributable to the protraction of mandibular molars.

With regard to the dentoalveolar changes, highly significant differences were observed between the two treatment strategies. Group FF had significant distal displacement of maxillary molar, whereas Group PE demonstrated mild mesial displacement, which is in agreement with the findings of Kizinger et al.^29^ However, the findings of the present study were not in consonance to those of Janson et al,^23^ who reported a mean anchorage loss of 4.10mm in extraction cases. Better maintenance of molar position in extraction group in the present study was attributable to the use of implant-anchored space closure. 

Effect on maxillary molar along the vertical plane was not significantly different between the groups. Findings of the present study regarding functional fixed treatment were in accordance with previously published studies.^30^

Groups PE and FF presented statistically significant difference for L6-VP and L6-GoMe at the posttreatment stage. A difference in sagittal positioning was consequent to protraction of mandibular molar to the extraction space. 

Maxillary incisors were significantly retroclined in Group FF (U1-ANS PNS, -5.73^o^), compared to Group PE (U1-ANS PNS, -4.09^o^), which is in consonance with previous studies^10^ ^,^ ^27^ but in disagreement with Weyrich and Lisson,^9^ who reported no difference in outcome between cases managed with premolar extraction and functional fixed treatment. The differences may be attributable to the type of functional appliance, as they used an Activator for treatment. The Group PE demonstrated significant reduction of U1-HP (-0.95mm) at the posttreatment stage. A difference in vertical positioning was consequent to intrusion/retraction mechanics for extraction space closure. Similar changes were reported by Upadhyay et al^27^ using an implant-anchored *en-masse* retraction. 

Despite cinching and lingual crown torque in the lower archwire during the functional phase, unfavorable labial movement of the mandibular incisors could only be minimized. Compared to Group PE (IMPA - 2.05^o^), subjects of Group FF (IMPA - 5.23^o^) had significantly proclined mandibular incisors, which is in consonance with previous studies,^13^ ^,^ ^35^ but in disagreement with Basciftci and Usumez,^34^ who demonstrated reduction of IMPA in extraction cases, contrary to the present findings. Despite the similar extraction pattern, difference in findings could be attributable to differences in anchorage mechanisms. 

Both treatment modalities reduced the overjet and the overbite to normal limits, with almost similar proportional correction. In Group PE, overall change of overjet and overbite was -4.23mm and -2.64mm, respectively; whereas Group FF demonstrated reduction of -4.73mm and -3.27mm, respectively. Similar changes were reported by Janson et al.^23^ Optimization of overjet in both groups was mainly due to dentoalveolar changes. In Group FF, it was mainly attributable to retroclination of maxillary incisors and proclination of mandibular incisors, whereas in Group PE, *en-masse* retraction as well as retroclination of maxillary incisor was attributed for the major change.

Treatment duration is one of the major factors influencing satisfaction among patients undergoing orthodontic treatment.^36^ There was no significant difference in overall treatment duration between the two groups, which is not in agreement with the findings of Janson et al,^37^ who reported premolar extraction treatment to be significantly shorter than non-extraction therapy. Difference in findings may be attributable to the use of extraoral headgear for correcting sagittal discrepancy. 

Mini-implants were used in Group PE in order to optimize the maxillary anterior teeth retraction and mandibular molar protraction. A recent systematic review^38^ refutes any significant advantages of using absolute anchorage in conjunction with fixed functional appliances, hence the same was not planned for Group FF.

Highly promising overall success rate of mini-implants (86.5%) was reported by a meta-analysis.^39^ In the present study, overall failure rate was 7.9%, i.e., only 7 implants of the total implants placed became loose, which is in agreement with previously published data.^39^ Four failures were reported on maxillary left side, two on the mandibular left side, and one on maxillary right side. There was no discontinuation of the treatment, as failed implants were retrieved and repositioned.

Forsus^TM^ failure rate of 37% has been reported in literature.^40^ Failure of Forsus^TM^ was encountered in three cases of the present study: Two of them reported breakage of the spring and one reported loss of slit crimp. Broken spring and lost crimp were replaced in the mentioned cases. Since one of these two cases had recurrent breakages of the spring, this case was excluded from the study. The higher failure rate of Bowman et al^40^ may be attributable to the varying experience level of the practitioners.

None of the participants asked to be removed from the study, reflecting acceptance of both treatment modalities. Premolar extraction treatment has limitations in terms of increased risk of root resorption,^41^ but offers the advantage of greater improvement of soft tissue profile. Functional treatment, on the other hand, results in insignificant profile change^10^ and significant mandibular incisor proclination, which is detrimental in terms of vertical alveolar bone loss.^42^

### LIMITATIONS

Ideally untreated Class II subjects should have been included in the present study as control. Although closely matched groups were included in the study, findings of the present study should be considered with caution, due to the short term observation period. Further studies with larger sample composition and long term follow-up are recommended to validate the findings. 

Lateral cephalograms have inherent limitations in terms of two dimensional (2D) evaluation of hard and soft tissues. Future studies using 3D technologies, such as color mapping and cone beam computed tomography, are strongly recommended. 

## GENERALIZABILITY

The results can be generalized to the average patient of most orthodontic clinical settings, as the present study was conducted in orthodontic departments of a government institute, based on broad inclusion criteria, and treatments were performed by experienced clinicians.

## CONCLUSION

The null hypothesis of this study was rejected. Management by extraction of premolars is a better treatment modality than functional treatment for managing skeletal Class II division 1 malocclusion in Class II subjects with moderate skeletal discrepancy, increased overjet, protruded maxillary incisors and protruded lips.

1. Both treatment modalities resulted in significant dentoalveolar changes; however, inclination of maxillary as well as mandibular incisors was better maintained in premolar extraction treatment.
2. Lip position relative to nose and chin, as well as nasolabial angle, improved significantly in maximum anchorage premolar extraction treatment cases.
3. Extraction treatment resulted in significant improvement of soft tissue profile.

## References

[1] Proffit WR, Fields HW, Moray LJ (1998). Prevalence of malocclusion and orthodontic treatment need in the United States estimates from the NHANES III survey. Int J Adult Orthodon Orthognath Surg.

[2] O'Brien K, Macfarlane T, Wright J, Conboy F, Appelbe P, Birnie D (2009). Early treatment for Class II malocclusion and perceived improvements in facial profile. Am J Orthod Dentofacial Orthop.

[3] Tadic N, Woods M (2007). Contemporary Class II orthodontic and orthopaedic treatment a review. Aust Dent J.

[4] Janson G, Mendes LM, Junqueira CH, Garib DG (2016). Soft-tissue changes in Class II malocclusion patients treated with extractions a systematic review. Eur J Orthod.

[5] Zierhut EC, Joondeph DR, Artun J, Little RM (2000). Long-term profile changes associated with successfully treated extraction and nonextraction Class II Division 1 malocclusions. Angle Orthod.

[6] Lim HJ, Ko KT, Hwang HS (2008). Esthetic impact of premolar extraction and nonextraction treatments on Korean borderline patients. Am J Orthod Dentofacial Orthop.

[7] Iared W, Koga da Silva EM, Iared W, Rufino Macedo C (2017). Esthetic perception of changes in facial profile resulting from orthodontic treatment with extraction of premolars a systematic review. J Am Dent Assoc.

[8] Pupulim DC, Henriques JFC, Janson G, Henriques FP, Freitas KMS, Garib D (2019). Comparison of dentoskeletal and soft tissue effects of Class II malocclusion treatment with Jones Jig appliance and with maxillary first premolar extractions. Dental Press J Orthod.

[9] Weyrich C, Lisson JA (2009). The effect of premolar extractions on incisor position and soft tissue profile in patients with Class II, Division 1 malocclusion. J Orofac Orthop.

[10] Zymperdikas VF, Koretsi V, Papageorgiou SN, Papadopoulos MA (2016). Treatment effects of fixed functional appliances in patients with Class II malocclusion a systematic review and meta-analysis. Eur J Orthod.

[11] Dolce C, McGorray SP, Brazeau L, King GJ, Wheeler TT (2007). Timing of Class II treatment skeletal changes comparing 1-phase and 2-phase treatment. Am J Orthod Dentofacial Orthop.

[12] Baysal A, Uysal T (2013). Soft tissue effects of Twin Block and Herbst appliances in patients with Class II division 1 mandibular retrognathy. Eur J Orthod.

[13] Oztoprak MO, Nalbantgil D, Uyanlar A, Arun T (2012). A cephalometric comparative study of class II correction with Sabbagh Universal Spring (SUS(2)) and Forsus FRD appliances. Eur J Dent.

[14] Konstantonis D, Vasileiou D, Papageorgiou SN, Eliades T (2018). Soft tissue changes following extraction vs nonextraction orthodontic fixed appliance treatment: a systematic review and meta-analysis. Eur J Oral Sci.

[15] Janson G, Junqueira CH, Mendes LM, Garib DG (2016). Influence of premolar extractions on long-term adult facial aesthetics and apparent age. Eur J Orthod.

[16] Kochar GD, Londhe SM, Shivpuri A, Chopra SS, Mitra R, Verma M (2021). Management of skeletal class II malocclusion using bimaxillary skeletal anchorage supported fixed functional appliances a novel technique. J Orofac Orthop.

[17] Sarver DM (2015). Interactions of hard tissues, soft tissues, and growth over time, and their impact on orthodontic diagnosis and treatment planning. Am J Orthod Dentofacial Orthop.

[18] Stahl F, Baccetti T, Franchi L, McNamara JA (2008). Longitudinal growth changes in untreated subjects with Class II Division 1 malocclusion. Am J Orthod Dentofacial Orthop.

[19] Antoun JS, Cameron C, Sew Hoy W, Herbison P, Farella M (2015). Evidence of secular trends in a collection of historical craniofacial growth studies. Eur J Orthod.

[20] Higgins JPT, Thomas J, Chandler J, Cumpston M, Li T, Page MJ (2019). Cochrane hand-book for systematic reviews of interventions.

[21] Janson G, Mendes LM, Junqueira CH, Garib DG (2016). Soft-tissue changes in Class II malocclusion patients treated with extractions a systematic review. Eur J Orthod.

[22] Bishara SE, Cummins DM, Jakobsen JR, Zaher AR (1995). Dentofacial and soft tissue changes in Class II, division 1 cases treated with and without extractions. Am J Orthod Dentofacial Orthop.

[23] Janson G, Castello Branco N, Aliaga-Del Castillo A, Henriques JFC, de Morais JF (2018). Soft tissue treatment changes with fixed functional appliances and with maxillary premolar extraction in Class II division 1 malocclusion patients. Eur J Orthod.

[24] Upadhyay M, Yadav S, Nagaraj K, Uribe F, Nanda R (2012). Mini-implants vs fixed functional appliances for treatment of young adult Class II female patients a prospective clinical trial. Angle Orthod.

[25] Lee YJ, Park JT, Cha JY (2015). Perioral soft tissue evaluation of skeletal Class II Division 1 a lateral cephalometric study. Am J Orthod Dentofacial Orthop.

[26] Kim K, Choi SH, Choi EH, Choi YJ, Hwang CJ, Cha JY (2017). Unpredictability of soft tissue changes after camouflage treatment of Class II division 1 malocclusion with maximum anterior re-traction using miniscrews. Angle Orthod.

[27] Upadhyay M, Yadav S, Patil S (2008). Mini-implant anchorage for en-masse retraction of maxillary anterior teeth a clinical cephalometric study. Am J Orthod Dentofacial Orthop.

[28] Wilmot JJ, Barber HD, Chou DG, Vig KW (1993). Associations between severity of dentofacial deformity and motivation for orthodontic-orthognathic surgery treatment. Angle Orthod.

[29] Kinzinger G, Frye L, Diedrich P (2009). Class II treatment in adults comparing camouflage orthodontics, dentofacial orthopedics and orthognathic surgery: a cephalometric study to evaluate various therapeutic effects. J Orofac Orthop.

[30] Franchi L, Alvetro L, Giuntini V, Masucci C, Defraia E, Baccetti T (2011). Effectiveness of comprehensive fixed appliance treatment used with the Forsus fatigue resistant device in Class II patients. Angle Orthod.

[31] Cozza P, Baccetti T, Franchi L, De Toffol L, McNamara JA (2006). Mandibular changes produced by functional appliances in Class II malocclusion: a systematic review. Am J Orthod Dentofacial Orthop.

[32] Bowman SJ, Johnston LE (2000). The esthetic impact of extraction and nonextraction treatments on Caucasian patients. Angle Orthod.

[33] Vaid NR, Doshi VM, Vandekar MJ (2014). Class II treatment with functional appliances a meta-analysis of short-term treatment effects. Semin Orthod.

[34] Basciftci FA, Usumez S (2003). Effects of extraction and nonextraction treatment on class I and class II subjects. Angle Orthod.

[35] Scott Conley R, Jernigan C (2006). Soft tissue changes after upper premolar extraction in Class II camouflage therapy. Angle Orthod.

[36] Pachêco-Pereira C, Pereira JR, Dick BD, Perez A, Flores-Mir C (2015). Factors associated with patient and parent satisfaction after orthodontic treatment a systematic review. Am J Orthod Dentofacial Orthop.

[37] Janson G, Barros SE, de Freitas MR, Henriques JF, Pinzan A (2007). Class II treatment efficiency in maxillary premolar extraction and nonextraction protocols. Am J Orthod Dentofacial Orthop.

[38] Elkordy SA, Aboelnaga AA, Fayed MM, AboulFotouh MH, Abouelezz AM (2016). Can the use of skeletal anchors in conjunction with fixed functional appliances promote skeletal changes A systematic review and meta-analysis. Eur J Orthod.

[39] Papageorgiou SN, Zogakis IP, Papadopoulos MA (2012). Failure rates and associated risk factors of orthodontic miniscrew implants a meta-analysis. Am J Orthod Dentofacial Orthop.

[40] Bowman AC, Saltaji H, Flores-Mir C, Preston B, Tabbaa S (2013). Patient experiences with the Forsus Fatigue Resistant Device. Angle Orthod.

[41] Proffit WR, Phillips C, Douvartzidis N (1992). A comparison of outcomes of orthodontic and surgical-orthodontic treatment of Class II malocclusion in adults. Am J Orthod Dentofacial Orthop.

[42] Matsumoto K, Sherrill-Mix S, Boucher N, Tanna N (2020). A cone-beam computed tomographic evaluation of alveolar bone dimensional changes and the periodontal limits of mandibular incisor advancement in skeletal Class II patients. Angle Orthod.

